# Speech Prosodies of Different Emotional Categories Activate Different Brain Regions in Adult Cortex: an fNIRS Study

**DOI:** 10.1038/s41598-017-18683-2

**Published:** 2018-01-09

**Authors:** Dandan Zhang, Yu Zhou, Jiajin Yuan

**Affiliations:** 1 College of Psychology and Sociology, Shenzhen University, Shenzhen, 518060 China; 2 Shenzhen Key Laboratory of Affective and Social Cognitive Science, Shenzhen University Shenzhen, Shenzhen, 518060 China; 3 The Laboratory for Affect Cognition & Regulation (ACRLAB), Key Laboratory of Cognition and Personality of Ministry of Education (SWU), Southwest University, Chongqing, 400715 China

## Abstract

Emotional expressions of others embedded in speech prosodies are important for social interactions. This study used functional near-infrared spectroscopy to investigate how speech prosodies of different emotional categories are processed in the cortex. The results demonstrated several cerebral areas critical for emotional prosody processing. We confirmed that the superior temporal cortex, especially the right middle and posterior parts of superior temporal gyrus (BA 22/42), primarily works to discriminate between emotional and neutral prosodies. Furthermore, the results suggested that categorization of emotions occurs within a high-level brain region–the frontal cortex, since the brain activation patterns were distinct when positive (happy) were contrasted to negative (fearful and angry) prosody in the left middle part of inferior frontal gyrus (BA 45) and the frontal eye field (BA8), and when angry were contrasted to neutral prosody in bilateral orbital frontal regions (BA 10/11). These findings verified and extended previous fMRI findings in adult brain and also provided a “developed version” of brain activation for our following neonatal study.

## Introduction

Perception of emotion in social interactions is important for inferring the emotional states and intentions of our counterparts. Communicated emotions expressed through face, body language and voice can be perceived and discriminated with multiple sensory channels^1^. However, while the literature on emotional perception has been well advanced with respect to the visual domain (e.g. see the review for facial expression studies^2^), the picture is less than complete for the auditory modality^3–5^. Instead of being a mere by-product of talking, speech prosody or affective melody (i.e. with frequency, intensity, rhythm, etc. as features) carried by human voices provides a rich source of emotional information that affects us consciously or nonconsciously^6^. (Note: In addition to speech prosody, there are other sound types conveying emotional information, such as environmental sounds, nonverbal expressions, singing and music^7^. This study only focused on speech prosodies.) A proper decoding of these emotional cues allows adaptive behavior in accordance with social context^8^.

With the advent of functional magnetic resonance imaging (fMRI), widespread cerebral networks have been suggested as neural bases of prosody decoding^6,7,9^. In particular, auditory temporal regions including the primary/secondary auditory cortex (AC) and the superior temporal cortex (STC)^8,10–15^, frontal areas such as the inferior frontal cortex (IFC)^16^ and orbital frontal cortex (OFC)^15,17^, insula^18^, and subcortical structures such as amygdala^19^ have been well acknowledged to be involved in the perception and comprehension of emotional prosody. Furthermore, a hierarchical model has been proposed for the processing of affective prosody^6,13,20^. The model suggests that (1) the extraction of acoustic parameters has been linked to voice-sensitive structures of the AC and mid-STC; (2) the posterior part of the right STC contributes to the identification of affective prosody by means of multimodal integration; and (3) further processing concerned with the evaluation and semantic comprehension of vocally expressed emotions is accomplished in the bilateral inferior frontal gyrus (IFG) and OFC^9,21^.

While the above-mentioned studies have formed a solid groundwork for the understanding of emotional prosody perception, rarely did these studies find activation differences between positive and negative prosody (for the only exception, see the fMRI study^22^ which found the activation was stronger for positive relative to negative prosody). Furthermore, although it is well known that the activation pattern of human brain is not the same for all emotions^23,24^, the question of how verbal expressions of different emotional categories elicit activation in temporal and frontal regions has been scarcely investigated^8^ (for the only exception, see the fMRI study by Kotz *et al*.,^15^ who found the bilateral superior middle frontal gyrus had enhanced activation for angry relative to neutral prosody while the left IFG had enhanced activation for happy relative to neutral prosody). In addition, fMRI studies on the effects of emotional sounds are unavoidably interfered with the gradient noise of the scanner so the fMRI-based results are necessary to be verified and complemented by a silent imaging method such as functional near-infrared spectroscopy (fNIRS)^25^. However, so far as we know, speech prosody has never been investigated using the fNIRS technique; and there are only three relevant fNIRS studies that examined nonverbal expressions or nonhuman sounds^25–27^. Therefore, the first aim of the present study was to provide an fNIRS-based knowledge of how speech prosodies of different emotional categories elicit activation in adult brain.

Another purpose of the current study was to provide a “developed version” of auditory response pattern to an on-going neonatal experiment in our lab. It is worth stressing that the use of fNIRS is irreplaceable for this purpose, because alternative methods such as fMRI and electroencephalography (EEG) cannot map the brain activation of conscious newborns with a high spatial resolution. To further make the results comparable between this study and the neonatal one, we required the adult subjects in this study to passively listen to affective prosodies because passive listening is the only feasible task for neonates (see neonatal studies^28,29^). Furthermore, since speech comprehension is largely immature in neonates’ undeveloped brain, we used semantically meaningless pseudosentences in these two studies so as to provide subjects with only prosody rather than both prosody and semantic information.

It was expected that while the voice-sensitive regions in the STC (including the primary/secondary AC) would be strongly activated by prosodies irrespective of emotional valence^5,8^, frontal regions such as IFC and OFC may have a crucial role in discrimination of verbal expressions of different emotional categories^7,9^. Since there is little knowledge of the brain activity associated with different categories of affective prosody, no hypothesis was made regarding the exact (if any) frontal areas that take part in decoding distinct affective cues embedded in happy, angry and fearful prosodies.

## Methods

### Participants

Twenty-two healthy subjects (12 females; age range = 18–24 years, 20.8 ± 0.4 years (mean ± std)) were recruited from Shenzhen University as paid participants. All subjects were right-handed and had normal hearing ability. Written informed consent was obtained prior to the experiment. The experimental protocol was approved by the Ethics Committee of Shenzhen University and this study was performed strictly in accordance with the approved guidelines.

### Stimuli

The emotional prosodies were selected from the Database of Chinese Vocal Emotions^30^. The database consists of “language-like” pseudosentences in Mandarin Chinese, which were constructed by replacing content words with semantically meaningless words (i.e. pseudowords) while maintaining function words to convey grammatical information. The structure of pseudosentences was equal (subject + predicate + object). The duration of each pseudosentence was approximately 1 to 2 sec.

Four kinds of emotional prosodies, i.e., fearful, angry, happy and neutral prosodies, were examined in this study. In order to construct four 15-sec segments for the four emotional conditions, we concatenated, separately, 11, 11, 8 and 9 pseudosentences of fearful, angry, happy and neutral prosodies. Among these pseudosentences, 6 were with the same constructions (but different emotions) across the four conditions. The mean speech rate of the four kinds of prosodies was 6.33, 6.53, 5.07 and 5.27 syllables/sec. The number of syllables for the four kinds of prosodies was 9.5 ± 1.0, 8.9 ± 1.8, 9.5 ± 1.6 and 8.8 ± 0.83 per sentence (mean ± std). All the selected emotional prosodies were pronounced by native Mandarin Chinese speakers (females), and the mean intensity was equalized. Before the experiment, the emotion recognition rate (mean = 0.80; select one emotion label from anger, happiness, sadness, fear, disgust, surprise, and neutral) and emotional intensity (5-point scale, mean = 3.1) were counterbalanced among the four conditions (the two measurements were from the database30). After the fNIRS recording, all the participants were required to classify each prosodic pseudosentences into one of four emotion categories. The mean recognition rate was 0.99 ± 0.04, 0.95 ± 0.08, 0.94 ± 0.08, 0.97 ± 0.06 for anger, fear, happy and neutral prosodies.

### Procedure

Sounds were presented via two speakers (R26T, EDIFIER, Dongguan, China) approximately 50 cm from the participants’ head. The speaker sound had a sound pressure level (SPL) of 60 to 70 dB (1353S, TES Electrical Electronic Corp., Taipei, Taiwan). The mean background noise level (without prosody presentation) was 30 dB SPL.

**Figure 1:**
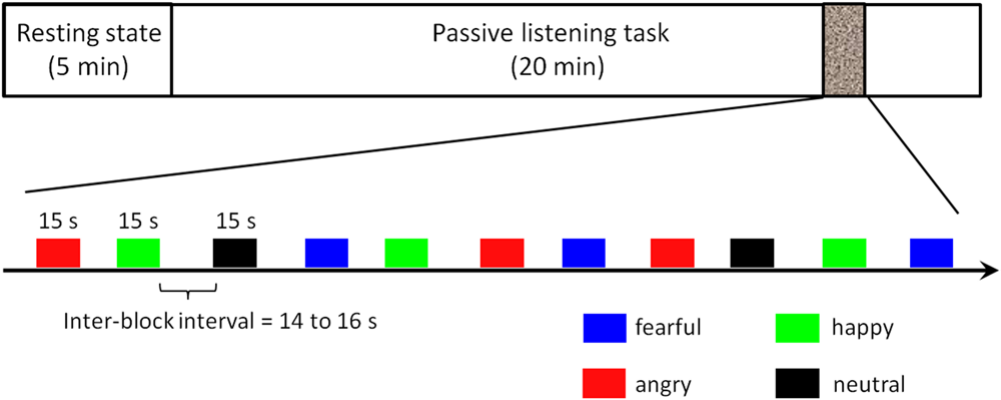
Schematic diagram of the timeline of the experiment.

The experiment lasted for 25 min (Fig. 1). Resting-state NIRS data were first recorded for 5 min (eyes opened), followed by a 20-min passive listening task. Each of the four 15-sec segments (corresponding to the four emotions) was repeated ten times. Thus there were 40 blocks in the study, which were presented in a random order. Inter-block interval (silent period) varied randomly between 14 and 16 sec.

### Data recording

**Figure 2:**
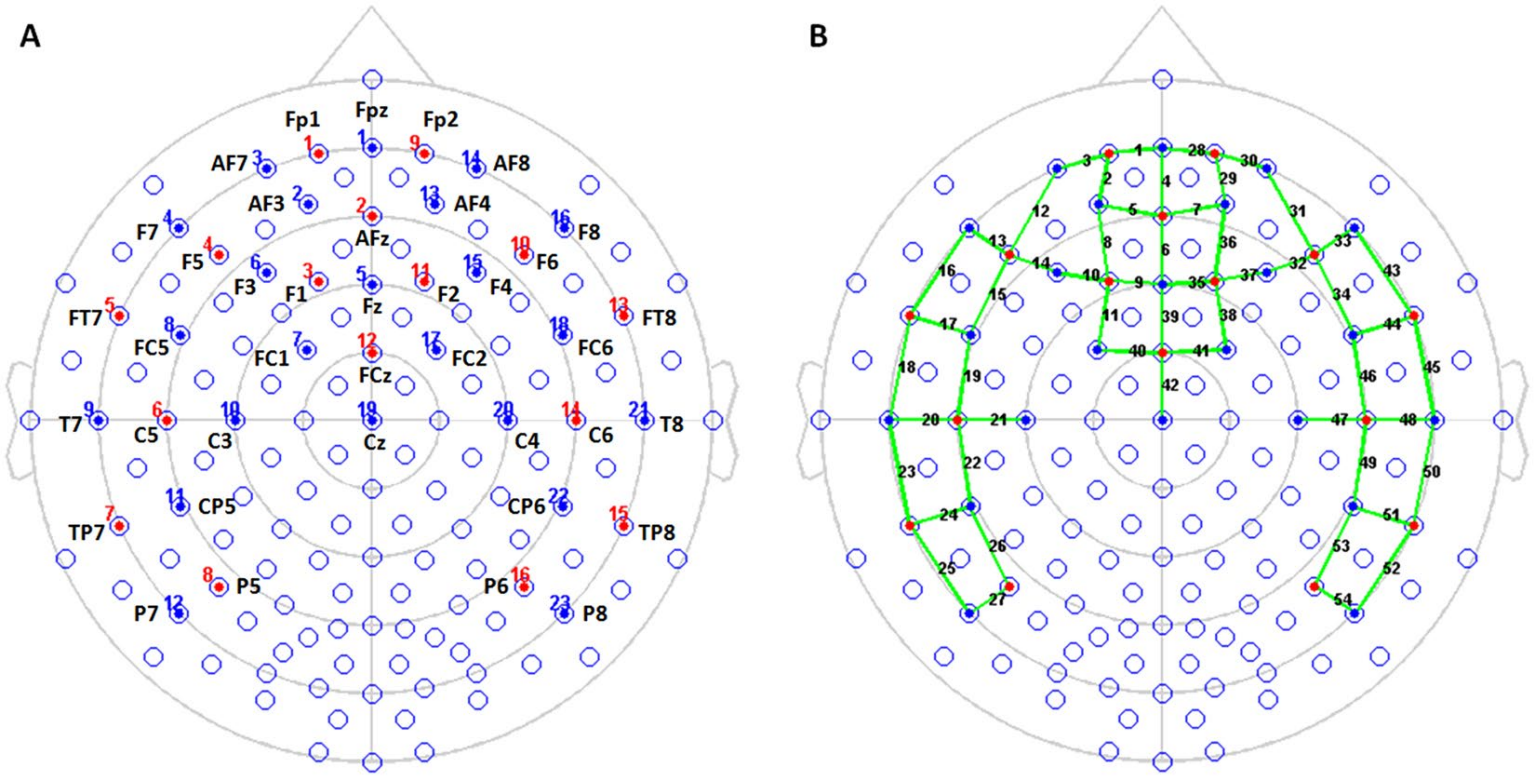
The locations of optodes and channels with respect to the EEG 10/5 system. (**A**), The locations of sources (red dots) and detectors (blue dots). (**B**), The 54 channels (green lines).

**Figure 3:**
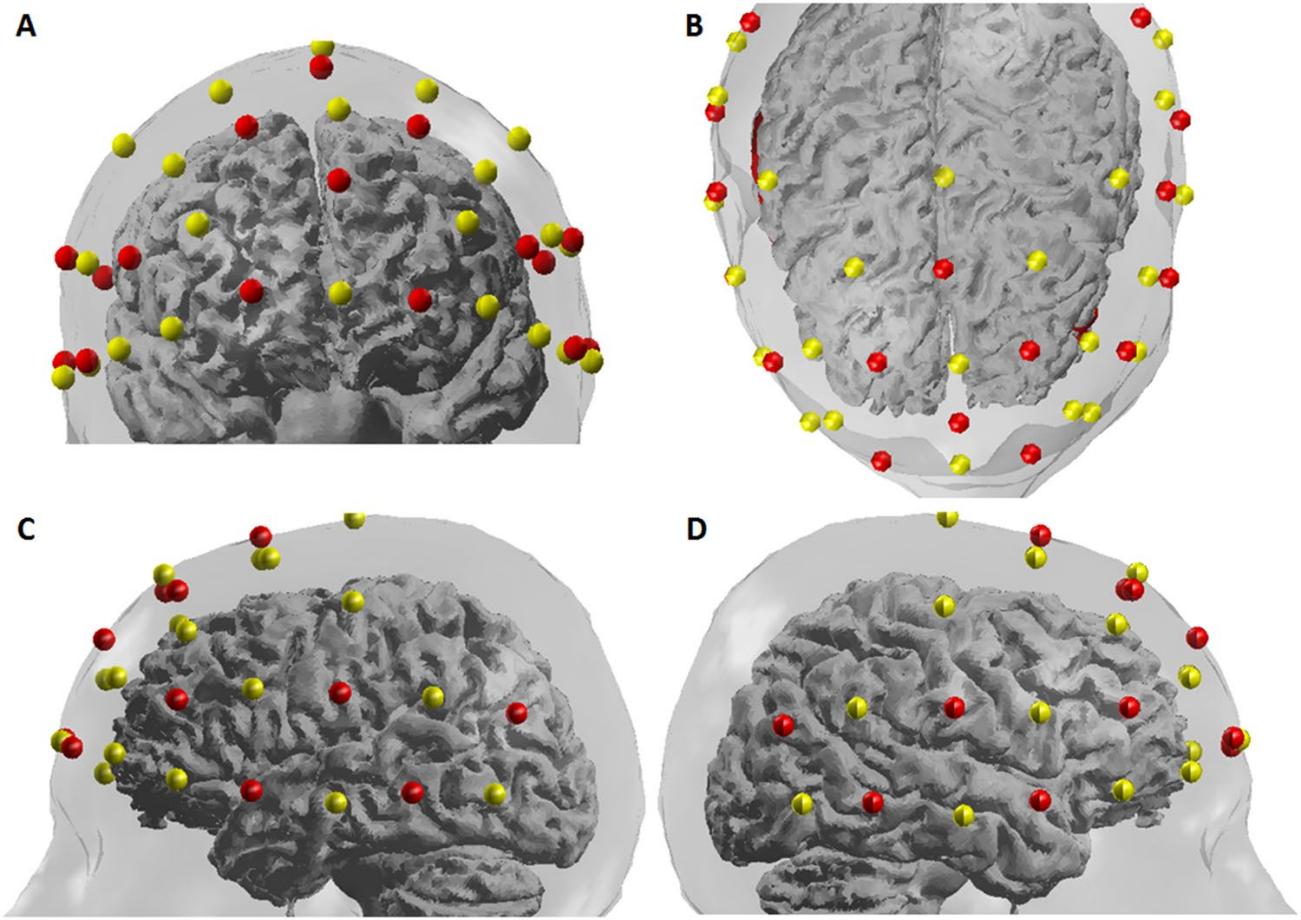
The locations of optical sources (red dots, n = 16) and detectors (yellow dots, n = 23) on a standardized 3D head. (**A**) front view. (**B**) Top view. (**C**) Left view. (**D**) right view.

The NIRS data were recorded in a continuous-wave mode with the NIRScout 1624 system (NIRx Medical Technologies, LLC. Los Angeles, USA), which consisted of 16 LED emitters (intensity = 5 mW/wavelength) and 23 detectors at two wavelengths (760 and 850 nm). Based on previous findings^6,7^, we placed optodes in the frontal and temporal regions of the brain, using a NIRS-EEG compatible cap (EASYCAP, Herrsching, Germany) with respect to the international 10/5 system (Figs. 2A and 3). There were 54 useful channels (Fig. 2B), where source and detector were at a mean distance of 3.2 cm (range = 2.8 to 3.6 cm) from each other. The data were continuously sampled with 4 Hz. Detector saturation never occurred during the recording.

To evaluate the cortical structures underlying NIRS channels, a Matlab toolbox NFRI (http://brain.job.affrc.go.jp/tools/)^31^ was used to estimate the NMI coordinates of optodes with respect to the EEG 10/5 positions. The locations of NIRS channels were defined at the central zone of the light path between each adjacent source-detector pair (Table 1).

**Table 1:** The MNI coordinates and cortical regions of the NIRS channels.

Channel		MNI coordinate			Brodmann area and anatomical label (percentage of overlap)*
		x	y	z	
1.	Fp1-Fpz	−10	68	−5	10 - Frontopolar area (0.62)
2.	Fp1-AF3	−25	66	4	10 - Frontopolar area (1.00)
3.	Fp1-AF7	−32	62	−8	10 - Frontopolar area (0.58)
					11 - Orbitofrontal area (0.42)
4.	AFz-Fpz	3	66	11	10 - Frontopolar area (1.00)
5.	AFz-AF3	−12	65	20	10 - Frontopolar area (1.00)
6.	AFz-Fz	2	54	38	9 - Dorsolateral prefrontal cortex (0.83)
7.	AFz-AF4	16	65	20	10 - Frontopolar area (1.00)
8.	F1-AF3	−24	55	31	9 - Dorsolateral prefrontal cortex (0.56)
					10 - Frontopolar area (0.44)
9.	F1-Fz	−10	44	48	8 - Includes Frontal eye fields (1.00)
10.	F1-F3	−30	45	39	9 - Dorsolateral prefrontal cortex (0.80)
11.	F1-FC1	−23	31	55	8 - Includes Frontal eye fields (0.85)
12.	F5-AF7	−46	48	0	10 - Frontopolar area (0.46)
					47 - Inferior prefrontal gyrus (0.34)
13.	F5-F7	−52	39	0	47 - Inferior prefrontal gyrus (0.62)
14.	F5-F3	−46	42	21	46 - Dorsolateral prefrontal cortex (0.83)
15.	F5-FC5	−56	27	16	45 - pars triangularis, part of Broca’s area (0.64)
16.	FT7-F7	−57	21	−13	38 - Temporopolar area (0.68)
17.	FT7-FC5	−61	8	2	22 - Superior Temporal Gyrus (0.61)
18.	FT7-T7	−66	−7	−14	21 - Middle Temporal gyrus (1.00)
19.	C5-FC5	−64	−2	24	6 - Pre-Motor and Supplementary Motor Cortex (0.67)
20.	C5-T7	−68	−17	8	42 - Primary and Auditory Association Cortex (0.51)
21.	C5-C3	−61	−16	41	6 - Pre-Motor and Supplementary Motor Cortex (0.55)
22.	C5-CP5	−66	−30	28	40 - Supramarginal gyrus, part of Wernicke’s area (0.73)
23.	TP7-T7	−69	−31	−9	21 - Middle Temporal gyrus (1.00)
24.	TP7-CP5	−67	−44	11	22 - Superior Temporal Gyrus (0.92)
25.	TP7-P7	−64	−55	−4	21 - Middle Temporal gyrus (0.58)
					37 - Fusiform gyrus (0.42)
26.	P5-CP5	−60	−56	28	40 - Supramarginal gyrus, part of Wernicke’s area (0.58)
27.	P5-P7	−58	−68	13	39 - Angular gyrus, part of Wernicke’s area (0.42)
28.	Fp2-Fpz	14	68	−5	10 - Frontopolar area (0.66)
29.	Fp2-AF4	28	66	4	10 - Frontopolar area (1.00)
30.	Fp2-AF8	35	63	−8	10 - Frontopolar area (0.63)
31.	F6-AF8	49	48	1	10 - Frontopolar area (0.45)
32.	F6-F4	48	42	22	46 - Dorsolateral prefrontal cortex (0.82)
33.	F6-F8	54	39	1	47 - Inferior prefrontal gyrus (0.56)
34.	F6-FC6	58	25	16	45 - pars triangularis, part of Broca’s area (0.69)
35.	F2-Fz	12	45	48	8 - Includes Frontal eye fields (0.98)
36.	F2-AF4	26	55	31	9 - Dorsolateral prefrontal cortex (0.57)
					10 - Frontopolar area (0.43)
37.	F2-F4	33	44	40	9 - Dorsolateral prefrontal cortex (0.72)
38.	F2-FC2	25	31	55	8 - Includes Frontal eye fields (0.84)
39.	FCz-Fz	1	30	57	8 - Includes Frontal eye fields (0.52)
					6 - Pre-Motor and Supplementary Motor Cortex (0.48)
40.	FCz-FC1	−12	16	64	6 - Pre-Motor and Supplementary Motor Cortex (1.00)
41.	FCz-FC2	14	17	64	6 - Pre-Motor and Supplementary Motor Cortex (1.00)
42.	FCz-Cz	1	1	69	6 - Pre-Motor and Supplementary Motor Cortex (1.00)
43.	FT8-F8	59	21	−12	38 - Temporopolar area (0.62)
44.	FT8-FC6	63	7	3	22 - Superior Temporal Gyrus (0.63)
45.	FT8-T8	67	−7	−12	21 - Middle Temporal gyrus (1.00)
46.	C6-FC6	66	−3	24	6 - Pre-Motor and Supplementary Motor Cortex (0.66)
47.	C6-C4	62	−16	40	6 - Pre-Motor and Supplementary Motor Cortex (0.57)
48.	C6-T8	70	−17	8	42 - Primary and Auditory Association Cortex (0.50)
49.	C6-CP6	67	−30	28	40 - Supramarginal gyrus, part of Wernicke’s area (0.78)
50.	TP8-T8	70	−30	−9	21 - Middle Temporal gyrus (0.98)
51.	TP8-CP6	68	−43	11	22 - Superior Temporal Gyrus (0.92)
52.	TP8-P8	64	−54	−4	37 - Fusiform gyrus (0.54)
					21 - Middle Temporal gyrus (0.46)
53.	P6-CP6	61	−56	28	40 - Supramarginal gyrus, part of Wernicke’s area (0.61)
54.	P6-P8	57	−67	13	39 - Angular gyrus, part of Wernicke’s area (0.54)

*The MNI coordinates were transformed to Talairach space^60,61^ (Laird *et al*.; Lancaster *et al*.) and looked up in a brain atlas^62^. One NIRS channel may be associated with several Brodmann areas. For the sake of brevity, here we only report the Brodmann areas with a percentage of overlap >0.40.

### Data preprocessing

The data were processed within the nirsLAB analysis package (v2016.05, NIRx Medical Technologies, LLC. Los Angeles, USA). Four out of the 22 datasets were deleted because the intensity (in volt) of more than 5 channels showed low values (the gain setting of the NIRx device >7). Thus a total of 18 datasets were analyzed in this study.

There are mainly two forms of movement artifacts in the NIRS data, i.e., transient spikes and abrupt discontinuities. First, spikes were smoothed by a semi-automated procedure which replaces contaminated data by linear interpolation. Second, discontinuities (or “jumps”) were automatically detected and corrected by the nirsLAB (std threshold = 5). Third, a band-pass filter (0.01 to 0.2 Hz) was applied to attenuate slow drifts and high frequency noises such as respiratory and cardiac rhythms. Then the intensity data were converted into optical density changes (ΔOD) (refer to the supplementary material for detailed procedure), and the ΔOD of both measured wavelengths were transformed to relative concentration changes of oxyhemoglobin and deoxyhemoglobin (Δ[HbO] and Δ[Hb]) by employing the modified Beer-Lambert law^32^. The source-detector distance of the first channel was 3.1 cm, and the exact distance of the other 53 channels was calculated by nirsLAB according to optode locations. The differential path length factor was assumed to be 7.25 for the wavelength of 760 nm and 6.38 for the wavelength of 850 nm^33^.

### Statistical analyses

Statistical significance of concentration changes was determined based on a general linear model of the canonical hemodynamic response function (parameters in nirsLAB = [6 16 1 1 6 0 32]), with a discrete cosine transformation used for temporal filtering (high-pass frequency cutoff = 128 sec). Although both Δ[HbO] and Δ[Hb] signals were obtained, we only chose Δ[HbO] to perform statistical analyses due to its superior signal-to-noise ratio relative to Δ[Hb]. When estimating beta, nirsLAB used a SPM-based algorithm (restricted maximum likelihood) to compute a least-squares solution to an overdetermined system of linear equations.

To statistically analyze the data, we first performed a one-way ANOVA on the beta values associated with Δ[HbO] (five levels: silence, neutral, fearful, angry and happy prosody), resulting in a thresholded (corrected *p* < 0.05) *F*-statistic map. Then six pairwise comparisons were followed up but only focusing on the significant channels revealed by the thresholded *F*-statistic map. This study was interested in the Δ[HbO] difference between (1) prosody and silence, (2) emotional and neutral prosody, (3) positive and negative prosody, (4) happy and neutral prosody, (5) angry and neutral prosody, (6) fearful and neutral prosody. The first two pairwise comparisons were used to verify and repeat the results of previous relevant studies; the last four pairwise comparisons were designed to explore activation differences between different emotional prosodies. The statistical results in individual channels were corrected for multiple comparisons across channels by the false discovery rate (FDR), following the Benjamini and Hochberg^34^ procedure implemented in Matlab (v2015b, the Mathworks, Inc., Natick, USA).

### Waveform visualization

In addition to statistic maps, we also displayed waveforms of Δ[HbO] and Δ[Hb] in the four emotional conditions (Figure S1 in supplementary material). This study considered Δ[HbO] and Δ[Hb] in a time window from −5 to 25 sec after the onset of emotional prosodies. The mean concentration of 5 sec immediately before each block was used as baseline (i.e., −5 to 0 sec; see also in other studies^35–37^).

## Results

### Main effect of experimental conditions

**Figure 4:**
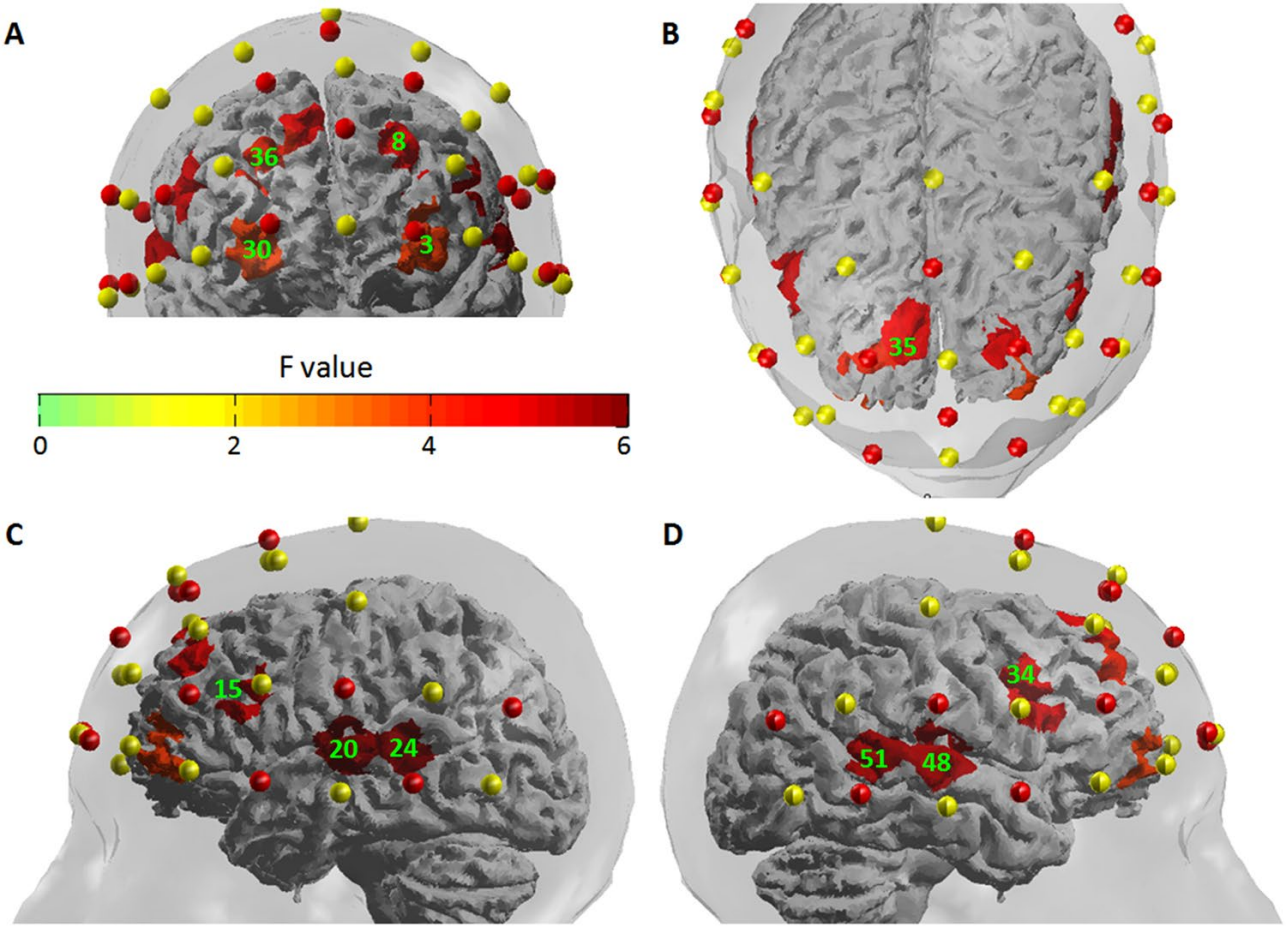
The *F*-statistic map showing brain regions that had different activation patterns across the five condition (silence, neutral, fearful, angry and happy prosody). Reported *F* values are thresholded by *p* < 0.05 (corrected for multiple comparisons using FDR). (**A**) front view. (**B**) top view. (**C**) left view. (**D**) right view. Green labels denote the number of channels.

The one-way ANOVA showed that 11 fNIRS channels (3, 8, 15, 20, 24, 30, 34–36, 48 and 51) had different activation patterns across the five experimental conditions (silence, neutral prosody and the three emotional prosody). The thresholded (corrected *p* < 0.05) *F*-statistic map is shown in Fig. 4, and the *F* values are summarized in Table 2. To measure the variation of beta values across individuals, the standard deviation of the beta values is reported in Table 3.

**Table 2:** Brain regions showed different activation patterns across experimental conditions (silence, neutral, fearful, angry and happy prosody).

Channel	Brodmann area (Talairach daemon) (percentage of overlap)	LPBA40 (percentage of overlap)	*F*(4,68)	*p*	corrected p*
24 TP7-CP5	22 - Superior Temporal Gyrus (0.92)	L superior temporal gyrus (0.62)	6.10	<0.001	0.012
20 C5-T7	42 - Primary and Auditory Association Cortex (0.51)	L superior temporal gyrus (0.69)	5.85	<0.001	0.012
51 TP8-CP6	22 - Superior Temporal Gyrus (0.92)	R middle temporal gyrus (0.60)	5.41	<0.001	0.012
		R superior temporal gyrus (0.40)			
48 C6-T8	42 - Primary and Auditory Association Cortex (0.50)	R superior temporal gyrus (0.89)	5.32	<0.001	0.012
34 F6-FC6	45 - pars triangularis, part of Broca’s area (0.69)	R inferior frontal gyrus (0.69)	5.05	0.001	0.012
15 F5-FC5	45 - pars triangularis, part of Broca’s area (0.64)	L inferior frontal gyrus (0.99)	5.04	0.001	0.012
35 F2-Fz	8 - Includes Frontal eye fields (0.98)	R superior frontal gyrus (0.96)	4.67	0.002	0.018
8 F1-AF3	9 - Dorsolateral prefrontal cortex (0.56)	L middle frontal gyrus (0.98)	4.54	0.003	0.020
	10 - Frontopolar area (0.44)				
36 F2-AF4	9 - Dorsolateral prefrontal cortex (0.57)	R middle frontal gyrus (1.00)	4.13	0.005	0.031
	10 - Frontopolar area (0.43)				
30 Fp2-AF8	10 - Frontopolar area (0.63)	R middle frontal gyrus (0.37)	3.91	0.006	0.035
3 Fp1-AF7	10 - Frontopolar area (0.58)	L middle frontal gyrus (0.49)	3.84	0.007	0.039
	11 - Orbitofrontal area (0.42)	L middle orbitofrontal gyrus (0.26)			

*p values were corrected for multiple comparisons using FDR.

**Table 3:** Standard deviation (across 18 subjects) of the beta values in the 54 channels.

Condition	1	2	3	4	5	6	7	8	9	10	11	12	13	14	15	16	17	18
fearful prosody	0.23	0.14	0.14	0.10	0.09	0.12	0.07	0.10	0.10	0.10	0.12	0.11	0.08	0.10	0.08	0.11	0.07	0.13
angry prosody	0.28	0.12	0.18	0.20	0.09	0.10	0.09	0.15	0.12	0.15	0.11	0.12	0.08	0.10	0.12	0.09	0.06	0.11
happy prosody	0.25	0.24	0.26	0.19	0.13	0.15	0.10	0.14	0.14	0.12	0.11	0.12	0.14	0.11	0.16	0.23	0.14	0.20
neutral prosody	0.28	0.14	0.20	0.20	0.11	0.16	0.12	0.16	0.13	0.16	0.15	0.12	0.15	0.13	0.16	0.18	0.12	0.15
silence	0.07	0.04	0.05	0.04	0.02	0.03	0.02	0.05	0.04	0.04	0.04	0.04	0.03	0.03	0.04	0.04	0.04	0.02
**Condition**	**19**	**20**	**21**	**22**	**23**	**24**	**25**	**26**	**27**	**28**	**29**	**30**	**31**	**32**	**33**	**34**	**35**	**36**
fearful prosody	0.14	0.14	0.14	0.09	0.12	0.10	0.11	0.13	0.13	0.18	0.12	0.10	0.09	0.07	0.07	0.07	0.08	0.15
angry prosody	0.09	0.11	0.12	0.12	0.12	0.12	0.19	0.16	0.18	0.24	0.13	0.24	0.13	0.10	0.12	0.09	0.10	0.16
happy prosody	0.13	0.11	0.11	0.09	0.13	0.08	0.13	0.14	0.15	0.22	0.25	0.25	0.19	0.10	0.10	0.13	0.14	0.20
neutral prosody	0.16	0.13	0.12	0.08	0.15	0.11	0.13	0.14	0.15	0.23	0.17	0.15	0.13	0.11	0.09	0.10	0.16	0.20
silence	0.04	0.04	0.05	0.03	0.04	0.04	0.05	0.05	0.06	0.06	0.04	0.04	0.03	0.03	0.02	0.03	0.04	0.07
**Condition**	**37**	**38**	**39**	**40**	**41**	**42**	**43**	**44**	**45**	**46**	**47**	**48**	**49**	**50**	**51**	**52**	**53**	**54**
fearful prosody	0.12	0.07	0.22	0.35	0.25	0.35	0.21	0.19	0.17	0.10	0.14	0.08	0.14	0.12	0.11	0.16	0.13	0.17
angry prosody	0.17	0.09	0.12	0.20	0.13	0.27	0.17	0.16	0.15	0.10	0.15	0.12	0.16	0.09	0.10	0.17	0.18	0.16
happy prosody	0.19	0.15	0.10	0.20	0.19	0.23	0.26	0.23	0.22	0.20	0.18	0.13	0.14	0.13	0.12	0.17	0.20	0.19
neutral prosody	0.18	0.12	0.18	0.16	0.11	0.14	0.27	0.21	0.29	0.16	0.16	0.16	0.17	0.11	0.09	0.15	0.16	0.15
silence	0.06	0.03	0.05	0.10	0.06	0.10	0.06	0.05	0.05	0.04	0.06	0.04	0.06	0.03	0.04	0.06	0.06	0.05

### Follow-up pairwise comparisons

#### *Contrast 1: prosody* > silence

First, we examined the brain regions associated with both emotional and neutral prosodies. The *t*-test showed that compared to the resting state (silence), four fNIRS channels had significantly enhanced activations in response to prosodies (Channel 20: *t*(17) = 4.54, *p* < 0.001, corrected *p* = 0.003; Channel 24: *t*(17) = 4.10, *p* < 0.001, corrected *p* = 0.007; Channel 34: *t*(17) = 3.28, *p* = 0.004, corrected *p* = 0.020; Channel 48: *t*(17) = 3.79, *p* = 0.002, corrected *p* = 0.010). The four channels correspond to brain regions of bilateral primary/secondary AC (Brodmann area (BA) 42), left posterior superior temporal gyrus (STG, BA 22), and right pars triangularis (middle IFG; BA 45). Among these brain areas, only the left primary/secondary AC (Channel 20) had convergent waveforms of Δ[HbO] and Δ[Hb] across the four conditions (Figure S1A). (Note: The time course of Δ[HbO] was different across the four conditions in the other four significant channels (e.g., see the waveforms at Channel 48, Channel 24 and Channel 34 in Figure S1) Furthermore, the activations within the primary/secondary AC showed leftward lateralization (paired-samples *t*-test: *t*(17) = 3.34, *p* = 0.004; Figure S1A).

In addition, there were another two channels showed significant deactivations (negative *t* values) in response to prosodies (Channel 8: *t*(17) = −5.84, *p* < 0.001, corrected *p* = 0.001; Channel 36: *t*(17) = −5.30, *p* < 0.001, corrected *p* = 0.002). The two channels correspond to brain regions of dorsolateral prefrontal cortex (DLPFC) and frontopolar prefrontal cortex (PFC).

#### *Contrast 2: emotional* > neutral prosody

Second, we examined the brain regions that were more activated for emotional compared to neutral prosodies. The *t*-test showed that compared to neutral prosodies, two channels had significantly enhanced activations in response to emotional prosodies, corresponding to brain regions of right posterior STG (BA 22, Channel 51; *t*(17) = 4.02, *p* < 0.001, corrected *p* = 0.035) and right primary/secondary AC (BA 42, Channel 48; *t*(17) = 3.46, *p* = 0.003, corrected *p* = 0.044). It is notable that while the main effect of prosodies (i.e. prosody contrasted to silence) had leftward lateralization in the posterior STG (paired-samples *t*-test: *t*(17) = 2.66, *p* = 0.017) and primary/secondary AC, the contrast of emotional and neutral prosodies within these areas showed rightward lateralization (AC: *t*(17) = −3.70, *p* = 0.002; STG: *t*(17) = −3.78, *p* = 0.001; Figure S1A and B).

#### *Contrast 3: positive* > negative prosody

Third, we examined the brain regions that were more activated for happy contrasted to fearful and angry prosody. The *t*-test showed that compared to negative prosody, two channels had significantly enhanced activations in response to happy prosody. The associated brain regions were left pars triangularis (middle IFG, BA 45, Channel 15; *t*(17) = 3.75, *p* = 0.002, corrected *p* = 0.039) and frontal eye fields (superior frontal gyrus, BA8, Channel 35; *t*(17) = 3.60, *p* = 0.002, corrected *p* = 0.039). It is notable that while the main effect of prosody (i.e. prosody contrasted to silence) had rightward lateralization in the middle IFG (paired-samples *t*-test: *t*(17) = −2.92, *p* = 0.010), the contrast of happy and fearful/angry prosody showed leftward lateralization (*t*(17) = 2.78, *p* = 0.013; Figure S1C).

#### *Contrast 4: happy* > neutral prosody

Fourth, we examined the brain regions that were more activated for happy contrasted to neutral prosody. The *t*-test showed that Channel 15 had significantly enhanced activations in response to happy prosody (*t*(17) = 4.12, *p* < 0.001, corrected *p* = 0.039). The associated brain regions were left pars triangularis (middle IFG, BA 45).

#### *Contrast 5: angry* > neutral prosody

Fifth, we examined the brain regions that were more activated for angry contrasted to neutral prosody. The *t*-test showed that two symmetrical channels had significantly enhanced activations in response to angry prosodies, corresponding to frontopolar and orbitofrontal areas (part of OFC, BA 10/11). However, the activation was not significant after multiple comparison correction (Channel 3: *t*(17) = 3.56, *p* = 0.002, corrected *p* = 0.070; Channel 30: *t*(17) = 3.74, *p* = 0.002, corrected *p* = 0.070; Figure S1D).

#### *Contrast 6: fearful* > neutral prosody

Finally, we examined the brain regions that were more activated for fearful contrasted to neutral prosody. No channels were significantly activated even before multiple comparison correction.

## Discussion

### The superior temporal cortex—decoding speech prosodies irrespective of emotional valence

The STC has been demonstrated to take a critical part in decoding vocal expressions of emotions (see meta-analysis^8^). (Note: The STC is comprised of STG, MTG, and the superior temporal sulcus^8^. The primary/secondary AC lies in the middle STG). While the lower-level structures of STC (i.e. the primary AC and mid-STC) analyze acoustic features in auditory expressions, the higher-level structures of STC integrate the decoded auditory properties and build up percepts of vocal expressions^7,21^. Consistent with this notion, the current study found that while speech prosodies activated the left primary AC (BA 42) most significantly when contrasting to silence, emotional prosodies activated the right STG (middle and posterior, BA 22/42) when contrasting to neutral prosodies. The right STG is the major structure of “emotional voice area“^38^, its anterior^20^, middle (or the primary and secondary AC)^6,9,17,39–42^ and especially posterior portion^6,9,13,17,41,43–45^ have been reported to show peak activations for emotional compared to neutral vocal expressions.

Our finding provides further evidence to clarify the lateralization of emotional prosody processing in the STC. It is observed that presentation of speech stimuli (i.e. prosody contrasted to silence) showed significant leftward lateralization in the primary/secondary AC and posterior STG, which is in line with the notion that the left hemisphere is better equipped for the analysis of rapidly changing phonetic representations in speech^15,17,21^. However, our data showed a strong right lateralization for affective prosody perception within the STC^7,15,17,25,44,46^, which is consistent with the finding that the right hemisphere is more sensitive to slow-varying acoustic profiles of emotions (e.g. tempo and pausing)^5,9,43,47^.

It is also worth noting that although we explored the cortex responses within six contrasts (i.e. follow-up pairwise comparisons), the STC showed significant activations only within the first two contrasts (i.e. prosody contrasted to silence and emotional contrasted to neutral prosodies). This result suggests that the STC may be implicated in general response to affective prosodies irrespective of valence or emotional categories, which is in line with many previous studies showing a U-shaped dependency between valence of prosodies and brain activation in the STC^14,18,42,48^.

In addition, we also observed two channels in frontal cortex (BA 9/10) showing deactivations in response to prosodies (contrasted to silence). This area located near but did not match with the default mode network (in particular, the medial prefrontal cortex) reported in fMRI studies. We guess this is due to technique limitations of the NIRS (see the Limitation subsection for details).

### The frontal cortex—discriminating speech prosodies of different emotional categories

One novel finding is that the left IFG (pars triangularis, BA 45) and the frontal eye field (BA8) were significantly activated for happy relative to fearful/angry prosodies. It has been reported that the pars triangularis of the IFG plays a critical role in semantic comprehension^21,49^. In this study, the finding of the higher tendency to semantically process happy relative to fearful and angry prosodies may be due to the positivity offset^50^, i.e., the participants felt less stressed in the happy than in the fearful or angry condition, so they were more motivated to comprehend happy prosodies though they were only required to passively listen. Since pseudosentences were used in the study, this potential semantic procedure may also activate the BA 8, which is involved in the management of uncertainty^51^. Previously three studies examined the neural bases of happy prosody processing. While Kotz *et al*.^15,22^ found happy (but not angry) relative to neutral prosodies activated left IFG, Johnstone *et al*.^52^ observed enhanced activation in right IFG for happy relative to angry prosodies. The incongruent lateralization of IFG activation may be due to the differences in stimuli, i.e., the participants in this study and in Kotz *et al*.^15,22^ only listened to speech prosodies but the participants listened to prosodies and watched congruent or incongruent facial expressions at the same time in Johnstone *et al*.^52^. The contrast of happy to neutral prosody in this study is consistent with the finding of Kotz *et al*.^15,22^.

Another interesting finding is the significant activation in bilateral OFC (BA 10/11) for angry contrasted to neutral prosody, which is almost consistent with the finding of Kotz *et al*.^15^. The OFC, which is a key neural correlate of anger^23^, plays an important role in conflict resolution and suppression of inappropriate behavior such as aggression^53,54^. Patients with bilateral damages of the OFC were found to be impaired with voice expression identification and had significant changes in their subjective emotional state^55^. Previous fMRI studies contrasting angry to neutral prosodies have reached different results: while some researchers believe that the bilateral frontal regions such as the OFC are always recruited regardless of implicit and explicit tasks^48,56^, some others found that only in explicit tasks the bilateral OFC responded to angry prosodies^39,41^. Considering the passive listening task in this study, we think the present finding supports the former opinion.

Surprisingly, no significant brain activations were found for fearful contrasted to neutral prosody. The result appears inconsistent with the notion of “the negativity bias” that favors the processing of fearful faces/pictures/words^50,57^. We propose that while visual emotional stimuli can be processed quickly, which helps individuals to initiate a timely fight-or-flight behavior; emotional prosodies communicate no biologically salient cues because their fine-grained features (e.g. pitch, loudness contour, and rhythm) evolve on a long time scale (i.e. longer than several seconds)^5^.

### Limitations

Finally, three limitations should be pointed out for an appropriate interpretation of the current result. First, the NIRS technique is only possible to measure brain activations on the surface of the cortex. Some brain regions that are highly involved in the processing of emotional prosodies (e.g. superior temporal sulcus, medial frontal cortex, ventral OFC and amygdala) are partially or totally untouchable. This may be the reason for the non-significant OFC activation after FDR correction in the follow-up pairwise comparison (angry > neutral prosody). Also, ventral frontal channels and channels across the midline of the frontal cortex (the influence of cerebrospinal fluid) did not show significant deactivation when prosody was contrasted to silence condition. Second, in order to provide comparable results for the on-going neonatal study, the adult subjects in the current study were required to passively listen to the prosodies (see also in other studies^12,27,42,58,59^). This task setting is suitable and may be the only feasible task for neonates, but may generate unnecessary voluntary perception and evaluation of emotional prosodies in adult’s brain. Since the activation pattern of the brain is task dependent^8^, a further adult study with a more rigorous task design (e.g., explicit/implicit tasks in some studies^6,20,48^) is needed to verify and complement the current findings. Third, this study did not use a set of pseudosentences that contained exactly the same words in the four emotional conditions, because the speech rate was different across emotions^30^ (i.e., although the structure of pseudosentences was equal, a small part of pseudosentences did not contain the same words across emotions). This issue, though inherent in affective prosody studies, may influence the results.

## Conclusion

In this study, we used fNIRS to investigate how speech prosodies of different emotional categories are processed in the cortex. Taken together, the current findings suggest that while processing of emotional prosodies within the STC primarily works to discriminate between emotional and neutral stimuli, categorization of emotions might occur within a high-level brain region–the frontal cortex. The results verified and extended previous fMRI findings in adult brain and also provided a “developed version” of brain activation for the following neonatal study.

## Electronic supplementary material

supplementary material
