# Supplementary material for: Speech Prosodies of Different Emotional Categories Activate Different Brain Regions in Adult Cortex: an fNIRS Study
Source: Sci Rep. 2018 Jan 9;8:218. doi: 10.1038/s41598-017-18683-2 (PMC5760650; doi:10.1038/s41598-017-18683-2)
Supplement: Supplementary file 1 — supplementary material [file 41598_2017_18683_MOESM1_ESM.pdf]

Speech Prosodies of Different Emotional Categories Activate Different Brain Regions in Adult Cortex: an fNIRS Study

Dandan Zhang[1,2,#], Yu Zhou[1,2,#], Jiajin Yuan[3,*]

1 College of Psychology and Sociology, Shenzhen University, Shenzhen 518060, China

2 Shenzhen Key Laboratory of Affective and Social Cognitive Science, Shenzhen University Shenzhen 518060, China

3 Faculty of Psychology, Southwest University, Chongqing 400715, China

# Both authors contributed equally to the study.

Running head: Emotional prosody processing in adult brain

The Authors have declared that there are no conflicts of interest in relation to the subject of this study.

* Corresponding author. Jiajin Yuan, The Laboratory for Affective Cognition and Regulation, Faculty of Psychology, Southwest University, NO.2 of Tiansheng Road, Beibei, Chongqing, China, 400715, E-mail: yuanjiajin168@126.com.

Supplementary material

The procedure for converting the data into optical density changes

The optical density (OD) is defined as follows,

$$OD = -log_{10}(I_{out} / I_{in})$$

where I_{out} represents the intensity of the detected light and I_{in} is of incident light. Practically, I_{in} is generally impossible measureable in continuous wave fNIRS. The change of optical density for a time point t_1 against an initial time t_0 is used instead to cancel out the incident light term, i.e.,

$$\Delta OD = OD_{t1} - OD_{t0} = -log_{10}(I_{out}^{t1} / I_{in}) + log_{10}(I_{out}^{t0} / I_{in}) = -log_{10} I_{out}^{t1} / I_{out}^{t0}$$

According the modified Beer-Lambert law, the changes of oxygenated hemoglobin Δ[HbO] and deoxygenated hemoglobin Δ[Hb] can be calculated from multiple (at least two) near-infrared light wavelength (λ) detections, i.e.,

$$\Delta OD^{\lambda_1} = (\varepsilon_{HbO}^{\lambda_1} \Delta C_{HbO} + \varepsilon_{Hb}^{\lambda_1} \Delta C_{Hb}) \cdot r \cdot DPF^{\lambda_1}$$
$$\Delta OD^{\lambda_2} = (\varepsilon_{HbO}^{\lambda_2} \Delta C_{HbO} + \varepsilon_{Hb}^{\lambda_2} \Delta C_{Hb}) \cdot r \cdot DPF^{\lambda_2}$$

Where $\varepsilon_{HbO}^{\lambda}$ and $\varepsilon_{Hb}^{\lambda}$ are the molar extinction coefficients of HbO and Hb respectively; *r* is the distance between detectors and sources; DPF is the differential path length factor accounting the increased light travel inside tissue due to scattering.

Figure

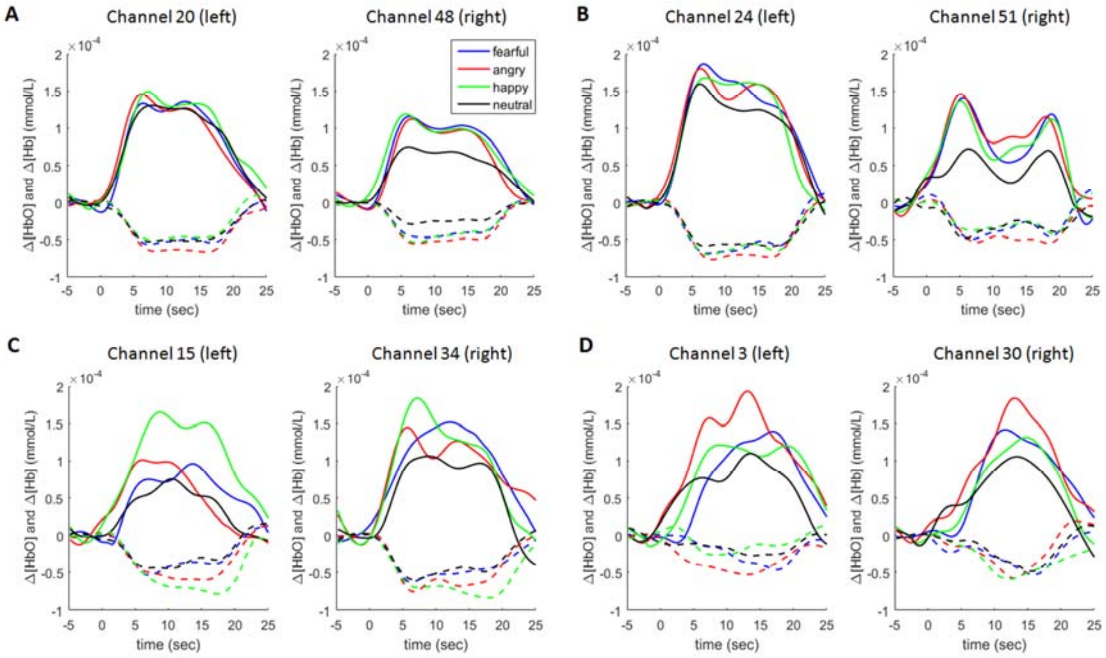

Figure S1. The time course of Δ[HbO] and Δ[Hb] in response to the four prosodies. The four subplots display the waveforms at A, bilateral primary auditory cortex (Channel 20 and Channel 48); B, bilateral posterior STG (Channel 24 and Channel 51); C, bilateral middle IFG (Channel 15 and Channel 34); and D, bilateral frontopolar and orbitofrontal areas (Channel 3 and Channel 30).
